# Facile synthesis and electrochemical performances of hollow graphene spheres as anode material for lithium-ion batteries

**DOI:** 10.1186/1556-276X-9-368

**Published:** 2014-07-28

**Authors:** Ran-Ran Yao, Dong-Lin Zhao, Li-Zhong Bai, Ning-Na Yao, Li Xu

**Affiliations:** 1 State Key Laboratory of Chemical Resource Engineering, Key Laboratory of Carbon Fiber and Functional Polymers (Beijing University of Chemical Technology), Ministry of Education, Beijing University of Chemical Technology, Beijing 100029, China; 2 State Grid Smart Grid Research Institute, Beijing 102211, China

**Keywords:** Lithium-ion batteries, Hollow graphene spheres, Electrochemical performance, Cycle performance

## Abstract

The hollow graphene oxide spheres have been successfully fabricated from graphene oxide nanosheets utilizing a water-in-oil emulsion technique, which were prepared from natural flake graphite by oxidation and ultrasonic treatment. The hollow graphene oxide spheres were reduced to hollow graphene spheres at 500°C for 3 h under an atmosphere of Ar(95%)/H_2_(5%). The first reversible specific capacity of the hollow graphene spheres was as high as 903 mAh g^-1^ at a current density of 50 mAh g^-1^. Even at a high current density of 500 mAh g^-1^, the reversible specific capacity remained at 502 mAh g^-1^. After 60 cycles, the reversible capacity was still kept at 652 mAh g^-1^ at the current density of 50 mAh g^-1^. These results indicate that the prepared hollow graphene spheres possess excellent electrochemical performances for lithium storage. The high rate performance of hollow graphene spheres thanks to the hollow structure, thin and porous shells consisting of graphene sheets.

**PACS:**

81.05.ue; 61.48.Gh; 72.80.Vp

## Background

Since the paper on freestanding graphene was published by Novoselov et al. [1], the preparation, structure, and property of graphene have attracted great attention owing to its particular quantum Hall effect, sensitivity, mechanical hardness, electrical conductivity, and so on [2-7]. Graphene is a two-dimensional one-atom-thick planar sheet of sp^2^ bonded carbon atoms, which is a basic building block for graphitic materials of all other dimensionalities. It is regarded as the ‘thinnest material in the universe’ with tremendous application potential. These attractive properties of graphene generate huge interest from different scientific communities in the possible implementation of graphene in different application fields such as biomedicine, reinforced composites, sensors, catalysis, energy conversion and storage device, electronics, and transparent electrodes for displays and solar cells [8].

Nowadays, lithium-ion batteries are widely used in various electronic devices, such as notebook computers, cellular phones, camcorders, electric vehicles, and electric tools due to their superior properties such as long cycle life, high energy density, no memory effect, and environmental friendliness. To meet the increasing demand for lithium-ion batteries with high reversible capacity and energy density, much effort has been made to develop new electrode materials or design novel structures of electrode materials [9-14]. Recently, graphene sheets as anode materials were investigated and exhibited large reversible capacity [15-19]; it has been demonstrated that the graphene sheets of ca. 0.7 nm thickness could provide the highest storage density (with a Li_4_C_6_ stoichiometry) by density of states calculations [20].

In this work, the hollow graphene oxide spheres (HGOSs) were fabricated directly from graphene oxide (GO) utilizing a water-in-oil emulsion technique, which were prepared from natural flake graphite by oxidation and ultrasonic treatment. The hollow graphene oxide spheres were reduced to hollow graphene spheres (HGSs) by heat treatment under a hydrogen atmosphere. Compared with the graphene sheets [21], the prepared HGSs possess better cycle and high rate performances for the lithium storage, which thanks to the hollow structure, thin and porous shells consisting of graphene sheets.

## Methods

GO nanosheets were prepared in two steps: the oxidation of flake natural graphite powder via a modified Hummers' method and ultrasonication. KMnO_4_ was employed as the oxidant to obtain graphite oxide. Firstly, 1 g of flake natural graphite powder with the mean diameter of 15 μm (provided by Dong Xin Electrical Carbon Co., Ltd., Chongqing, China) was added to 23 mL of cooled (0°C) concentrated H_2_SO_4_. Then, 3 g of KMnO_4_ was added gradually with stirring and cooling, so that the temperature of the mixture was maintained below 10°C. The mixture was then stirred at 35°C for 30 min. After this, 46 mL of distilled water was slowly added to cause an increase in temperature to 98°C, and the mixture was maintained at that temperature for 15 min. The reaction was terminated by adding 140 mL of distilled water followed by 10 ml of 30% H_2_O_2_ solution. The suspension was then repeatedly centrifuged and washed twice with 5% HCl solution and then repeatedly with water until sulfate could not be tested with barium chloride. The collected precipitate was dispersed in 450 mL water and sonicated for 2 h. Then, the suspension was separated into the supernatant liquor and a golden colored residue by centrifugation at 5,000 rpm for 10 min. The supernatant was centrifuged again at 15,000 rpm for 5 min to remove the suspended substance. The precipitate was ultrasonicated, collected, and dried in a vacuum oven at 60°C; thus, GO nanosheets were obtained.

GO nanosheets of 0.1 g were dispersed into aqueous ammonia (20 mL, pH = 12) through agitation and were stirred at 30°C for 1 h to obtain the GO nanosheet suspension. Then, the suspension was slowly poured into hot olive oil (provided by Asceites Del Sur-coosur, Seville, Spain; the acidity is <0.4%, and the saturated fat, polyunsaturated fat, and monounsaturated fat are 14, 9, and 77 wt%, respectively) preheated to 90°C and intensely stirred for 30 min at 90°C. Subsequently, with the formation of a water-in-oil emulsion, the viscosity of the emulsion rapidly increased with the appearance of a golden foam. Half an hour later, when the bath temperature was increased to 95°C, the viscosity decreased gradually. With the intensive stirring, water was gradually separated from the oil. In the meantime, emulsion turned clear as olive oil. Finally, the emulsion system was cooled to room temperature. The HGOSs were obtained by centrifugation, washing, and drying. The HGOSs were reduced to HGSs at 500°C for 3 h under an atmosphere of Ar(95%)/H_2_(5%).

The products were characterized by X-ray diffraction (XRD) on a Rigaku D/max-2500B2+/PCX system (Rigaku, Beijing, China) using Cu/K radiation (*λ* = 1.5406 Å) over the range of 5 to 90° (2θ) at room temperature. The morphologies of the samples were observed by scanning electron microscope (SEM, Hitachi S-4700, Hitachi, Ltd, Chiyoda-ku, Japan). The information of functional groups was measured by Fourier transform infrared spectroscopy instrument (FTIR, Nicolet Nexus 670, Thermo Fisher Scientific, Shanghai, China).

The electrochemical performances of the HGSs as anode materials for lithium-ion batteries were measured with the coin-type cells. The lithium sheets were used as both reference and counter electrodes, and composite electrodes comprising active mass (HGSs, 85 wt%), carbonaceous additive (acetylene black, 5 wt%), and poly(vinylidene difluoride) (PVDF, 10 wt%) binder were used as working electrodes. The thickness and density of electrode are 50 μm and 1.95 mg cm^-2^, respectively. One molar LiPF_6_ solution in a 1:1 (volume) mixture of ethylene carbonate (EC) and dimethyl carbonate (DMC) from Merck & Co., Inc. (Whitehouse Station, NJ, USA) was used as electrolyte. The Celgard 2400 microporous polypropylene film provided by Jimitek Electronic (Shenzhen, China) Co. Ltd was used as separator. The coin-type cells were galvanostatically discharged (Li insertion) and charged (Li extraction) in the voltage range from 0.01 to 3.50 V vs. Li/Li^+^ at the different current densities. Electrochemical impedance spectroscopy measurements of the electrodes were carried out on an electrochemical workstation (Princeton VersaSTAT3-200, Princeton Applied Research, Oak Ridge, TN, USA) using the frequency response analysis. The impedance spectra were obtained by applying a sine wave with amplitude of 5.0 mV over the frequency range from 100 kHz to 0.01 Hz.

## Results and discussion

**Figure 1:**
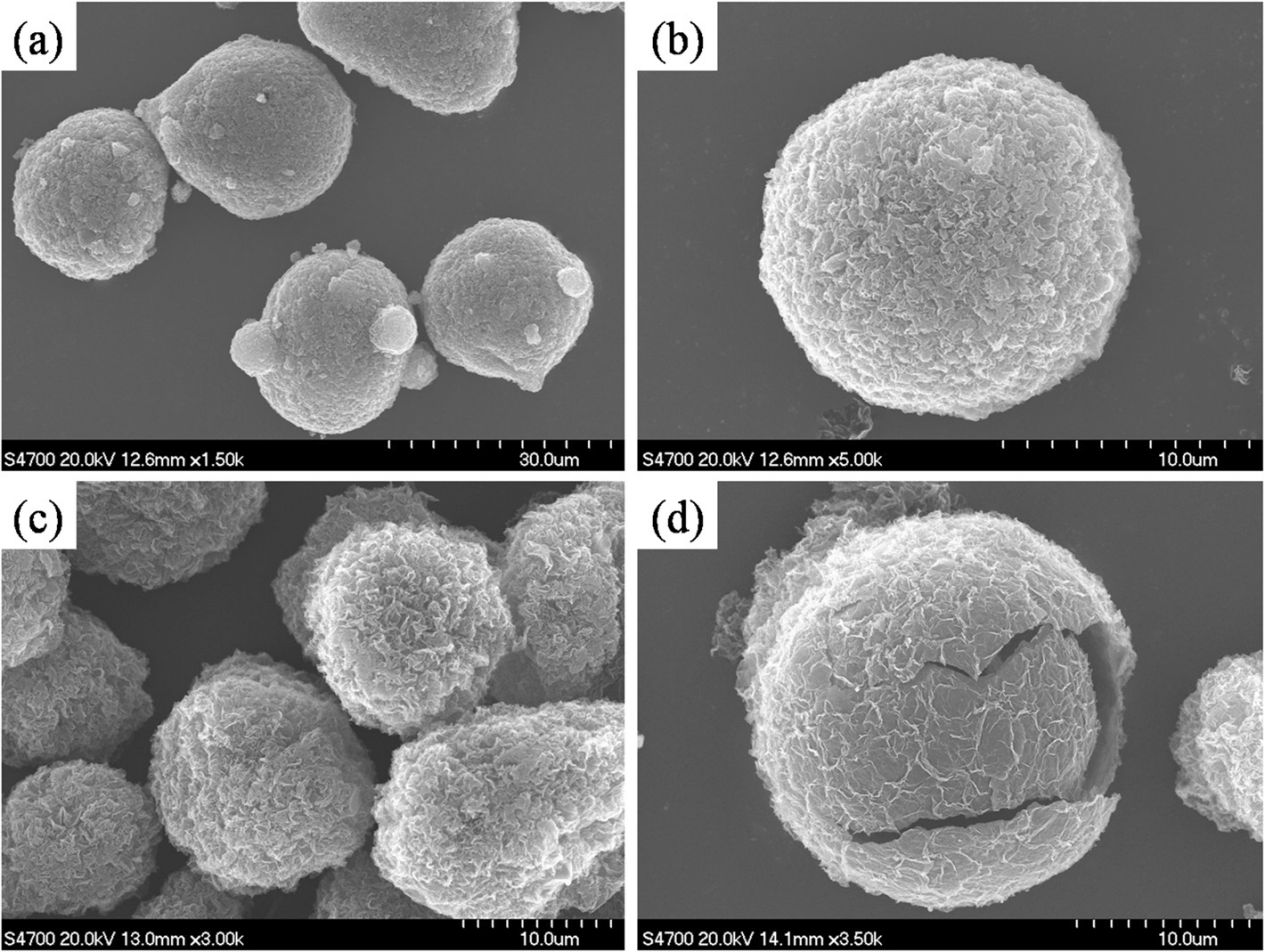
**SEM images of HGOSs (a and b) and HGSs (c and d).**

The morphology and structure of HGOSs and HGSs were characterized by SEM, and their images are shown in Figure 1. SEM images in Figure 1 exhibit the hollow structures of HGOSs and HGSs. In particular, some spheres collapse after heat treatment as shown in Figure 1d. The SEM images in Figure 1c,d show that HGSs hold a compact and hollow microstructure, distinct from the laminar structure of bulk graphite oxide and paper-like texture of graphene nanosheets. From Figure 1a, it is observed that some small holes and protuberances emerge on the surface of microspheres, which is assigned to the removal of water and will be discussed in detail later. An unambiguously broken sphere reveals that the interior is hollow, and the thickness of the wall is approximately 1 μm (Figure 1d). The continuous and smooth cross section implies that the adjacent graphene nanosheets possess a close connection.

**Figure 2:**
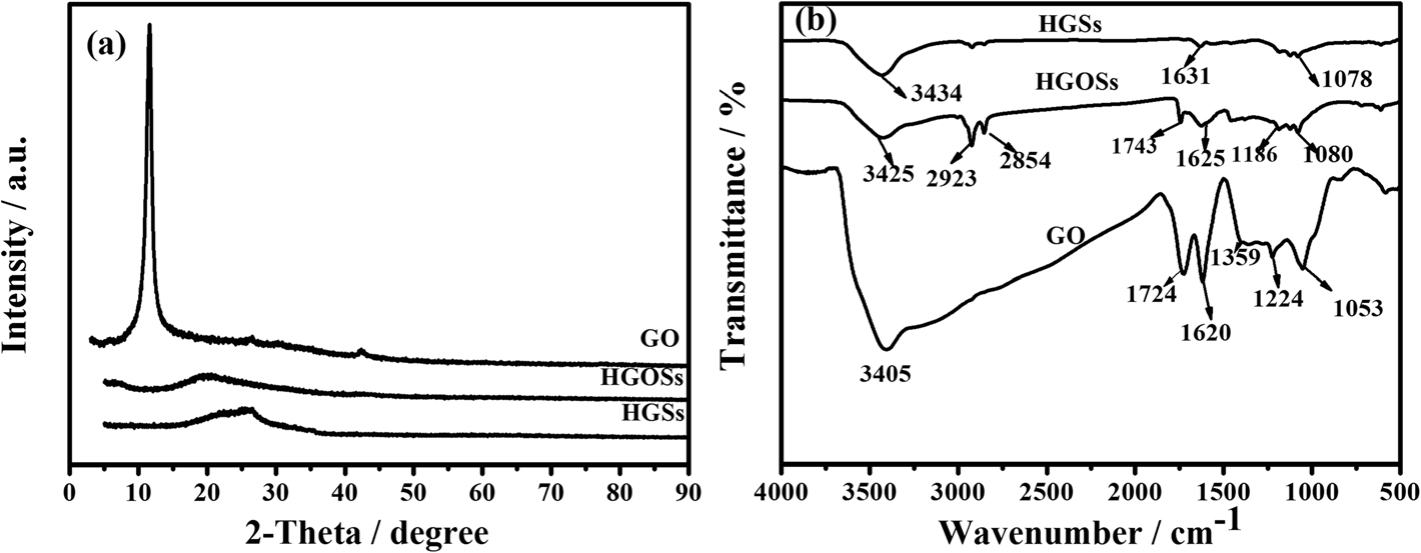
**XRD patterns (a) and FTIR spectra (b) of GO**, **HGOs, and HGSs.**

The structural changes from GO to HGSs were investigated by XRD measurement, and the patterns are shown in Figure 2a. After oxidation, the (002) peak of graphite disappears, and an additional peak at 11.56° is observed, which is corresponding to the (001) diffraction peak of GO. The *d*-spacing of GO increased to 0.765 nm from 0.335 nm of graphite, which is ascribed to the oxide-induced O-containing functional groups and inserted H_2_O molecules [17,22] that can be confirmed by FTIR. After an emulsion process, it is observed that the strong (001) diffraction peak of HGOSs is weakened, possibly because the partial oxygen-containing groups and bound moisture are consumed through reaction with ammonia and the following water removal process. In the meantime, the (002) diffraction peak was partially recovered, suggesting that the graphene layers rearranged during the emulsion process. After heat treatment, the diffraction peak of GO disappears, indicating that HGOSs has successfully reduced to HGSs. Figure 2b shows FTIR spectra of GO, HGOs, and HGSs. For GO, the peak at 3,405 cm^-1^ can be attributed to O-H stretching vibrations of adsorbed water molecules and structural OH groups, and the peak at 1,619 cm^-1^ can be attributed to O-H bending vibrations. The presence of carboxyl and epoxy functional groups can also be detected at around 1,724 and 1,224 and 1,053 cm^-1^, respectively [17,22]. These evidences indicate that during the oxidation process of graphite with KMnO_4_ in the concentrated sulfuric acid, the original extended conjugated π-orbital system of graphite were destroyed, and oxygen-containing functional groups were inserted into carbon skeleton. Therefore, it is reasonable to believe that GO nanosheets should be regarded as ‘amphiphilic molecules’ and perform a surfactant-like function in a water/oil emulsion system [23]. Due to the introduction of acid groups on the edge sites and basal planes of graphene sheets, GO nanosheets are well-dispersed in alkali solution. On the basis of the experimental results, a scheme is presented to describe the formation process of nano HGOSs self-assembled by water/oil emulsion. It includes four steps: (1) the delamination of graphite after intensive oxidation; (2) the homogeneous mixture of GO nanosheets and aqueous ammonia; (3) the formation of a water-in-oil emulsion containing GO nanosheets; (4) and the removal of water and the separation of HGOSs from olive oil. When aqueous ammonia containing GO nanosheets is mixed with olive oil by mechanical agitation, a water-in-oil system is formed. GO nanosheets were supported by the water-in-oil interface and self-assembled around water droplets under the assistance of ammonia. With the removal of aqueous ammonia, the GO nanosheets stacked and condensed at the water-in-oil interface and finally formed a shell structure around the soft template.

After a thermal treatment in H_2_, these functional groups derived from the intensive oxidation were eliminated, which can be proved by the disappearance of the peaks at 1,724, 1,619, 1,224, and 1,053 cm^-1^ while an appearance of a new peak at 1,631 cm^-1^ (Figure 2b) reflecting the skeletal vibration of graphene sheets [15,22]. These evidences indicate that HGOSs have been successfully reduced to HGSs by heat treatment.

**Figure 3:**
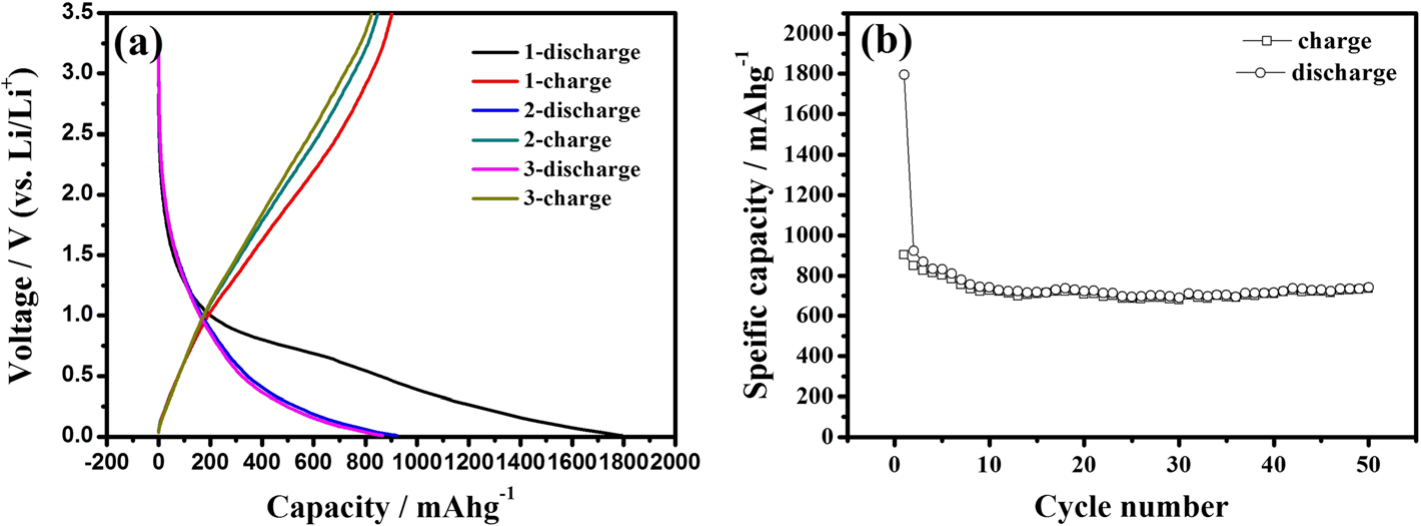
**First three discharge/charge profiles (a) and cycle performances (b) of HGSs at the current density of 50 mA g**^**-** **1**^**.**

**Figure 4:**
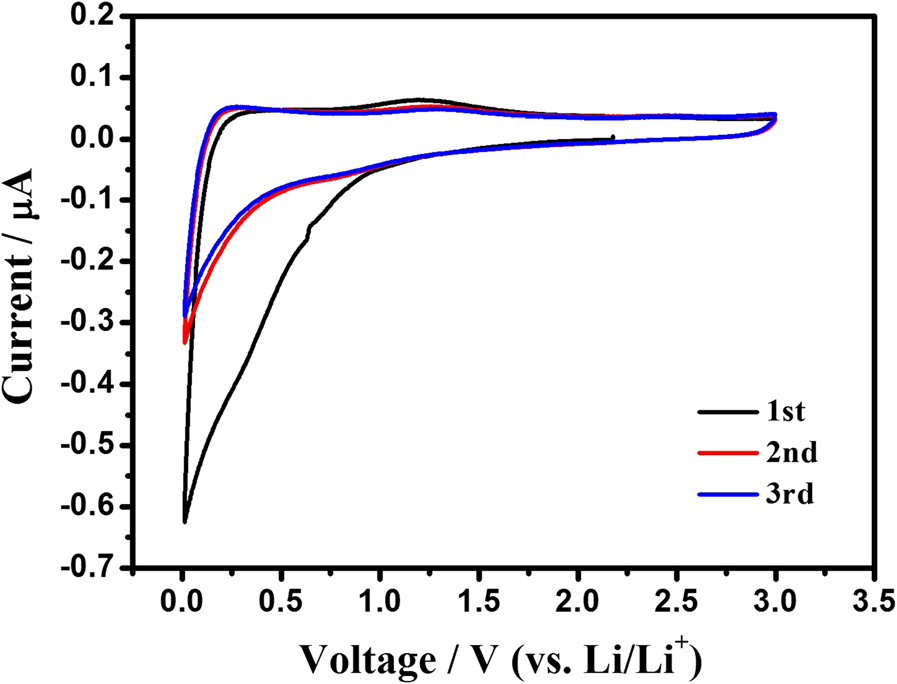
**Cyclic voltammograms (CV) of HGSs.**

Figure 3a shows the first three charge–discharge voltage profiles of HGS electrodes vs. Li/Li^+^ at the current density of 50 mA g^-1^. The first charge curve for HGSs has plateaus at about 0.7 V representing the solid electrolyte interface (SEI) film formation and the generation of irreversible capacity. From the second cycle, the charge/discharge curve of HGS slope without distinguishable plateaus, which can be attributed to the smaller crystallite structure, high specific surface area [24], and disorganized graphene stack [15,16]. For HGSs, the first-cycle discharge and charge capacities are 1,794 and 902 mA h g^-1^, respectively. Obviously, the reversible capacity of HGSs is much higher than that of previously reported graphene nanosheets (672 mA h g^-1^ at a current density of 0.2 mA cm^-2^) [15]. The possible reason is that the larger surface area and curled morphology of HGSs with fewer layers can provide more lithium insertion active sites, such as edge-type sites and nanopores [25]. The possible reversible reaction of Li with the residual H in the HGSs and faradaic contribution are also favorable to the large reversible capacity [26]. It is well known that the disordered carbons can yield higher capacity values than graphite [27], and the graphene can be considered as a very disordered carbon. It should be noted that the HGS electrodes exhibit a broad electrochemical window (0.01 to 3.5 V) as a function of lithium capacity and the large voltage hysteresis between discharge and charge voltage curves, which is different from graphite and similar to the nongraphitic carbons [21,24-28]. The large voltage hysteresis is related to active defects in the disordered graphene nanosheets. The reaction of Li with the active defects in discharge processes occurs at low voltages, but the break of the relatively strong bonds of Li with the defects in charge processes requires higher voltages, thus resulting in the large voltage hysteresis [19]. The reversible specific capacity of the prepared HGSs reduced to 848 mA h g^-1^ in the second cycle, but it was still maintained at 741 mA h g^-1^ in the fifth cycle. This evidence indicates that the prepared HGSs exhibited stable cyclic performance from the second cycle because of the formed stable SEI film during the first discharge process. The cyclic voltammograms (CV) of the prepared HGSs are shown in Figure 4. The shape of the CV curves matches well with the discharge/charge profiles (Figure 3a).

**Figure 5:**
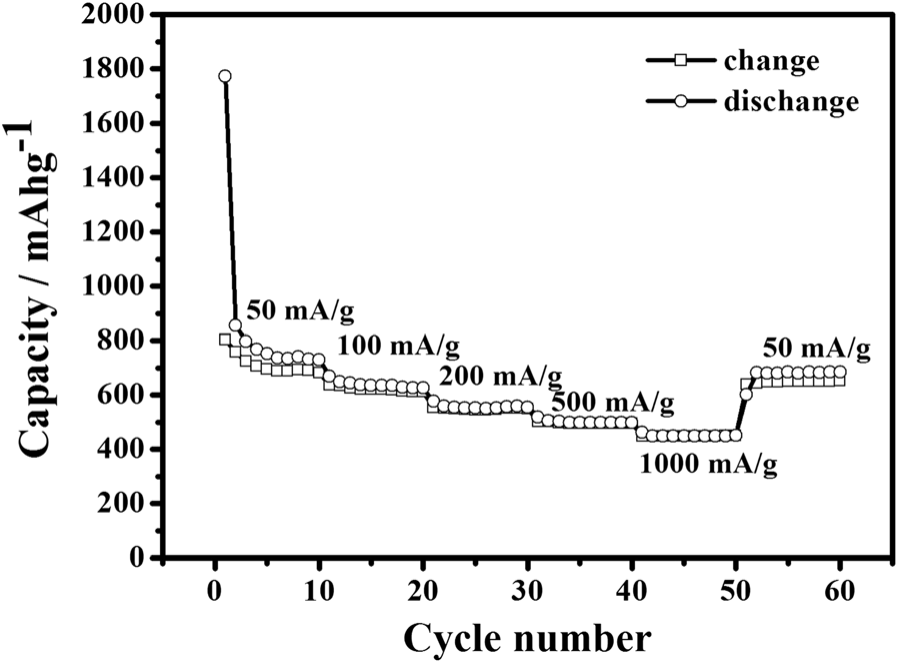
**Cycle performance of HGSs at the current densities from 50 mA g**^**-** **1**^**to 1,000 mA g**^**-** **1**^**.**

Cycle performance of HGSs at different current densities of 50 mA g^-1^, 100 mA g^-1^, 200 m mA g^-1^, 500 m mA g^-1^, and 1,000 mA g^-1^ are shown in Figure 5. After 60 cycles, it was found that the reversible capacity was still maintained at 652 mA g^-1^ for HGSs. These results demonstrated that the prepared HGSs have an intensive potential as a candidate of anode materials with high reversible capacity, good cycle performance, and high rate discharge/charge capability.

**Figure 6:**
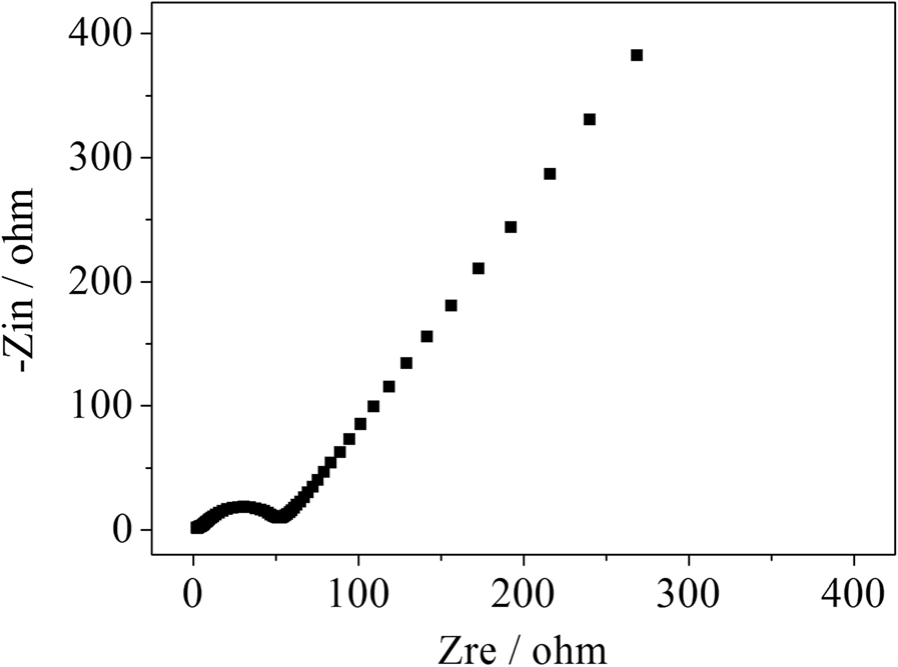
Nyquist impedance plots for HGS electrode.

To investigate the kinetics of electrode process of HGS electrode, its Nyquist complex plane impedance plots are presented in Figure 6. The high-frequency semicircle is corresponded to formation of SEI film and/or contact resistance, the semicircle in medium-frequency region is assigned to the charge-transfer impedance on electrode/electrolyte interface, and the inclined line at an approximate 45° angle to the real axis corresponds to the lithium-diffusion process within carbon electrodes [14,15]. Electrochemical impedance spectrum measurement (Figure 6) shows that the charge-transfer resistance of the HGS electrode is very low (ca. 28.1 Ω) after a simulation using an equivalent circuit (details referred to in [29]), indicating the formation of a better conductive network in the HGS electrode.

## Conclusions

The HGSs have been successfully fabricated from GO nanosheets utilizing a water-in-oil emulsion technique and thermal treatment. The electrochemical performance testing showed that the first reversible specific capacity of the HGSs was as high as high as 903 mAh g^-1^ at a current density of 50 mAh g^-1^. After 60 cycles at different current densities of 50 mA g^-1^, 100 mA g^-1^, 200 m mA g^-1^, 500 m mA g^-1^, and 1,000 mA g^-1^, the reversible specific capacity was still maintained at 652 mA g^-1^ at the current density of 50 mA g^-1^, which indicated that the prepared HGSs possess a good cycle performance for the lithium storage. The high rate performance of HGSs thanks to the hollow structure, thin and porous shells consisting of graphene sheets.

## Abbreviations

HGOSs: hollow graphene oxide spheres; GO: graphene oxide; HGSs: hollow graphene spheres; XRD: X-ray diffraction; SEM: scanning electron microscope; FTIR: Fourier transform infrared; SEI: solid electrolyte interface; CV: cyclic voltammograms.

## Competing interests

The authors declare that they have no competing interests.

## Authors’ contributions

RRY designed parts of the experiments and sample preparations and drafted the manuscript. DLZ is the corresponding author and provided a great help for experimental designs. LZB, NNY, and LX took part in sample preparation and characterizations and discussed the results. All authors have read and approved the final manuscript.
